# Monobloc vs. Modular Radial-Head Arthroplasty for Complex Elbow Trauma: Long-Term Follow-Up and Comparative Evaluation

**DOI:** 10.3390/jpm14091006

**Published:** 2024-09-21

**Authors:** Shai Factor, Ron Gurel, Daniel Tordjman, Gilad Eisenberg, Tamir Pritsch, Yishai Rosenblatt

**Affiliations:** Hand Surgery Unit, Division of Orthopedics, Tel Aviv Sourasky Medical Center, Affiliated to the Faculty of Medicine, Tel Aviv University, Tel Aviv 6997801, Israel

**Keywords:** radial-head fracture, radial-head arthroplasty, prosthesis, modular, monobloc, long-term outcomes

## Abstract

**Introduction:** Mason Type 3 radial-head fractures are typically treated with open reduction and internal fixation (ORIF) or radial-head arthroplasty (RHA). Prosthetic options include traditional monobloc implants and newer modular implants designed to match patient anatomy. While short- and medium-term outcomes of metallic RHA are generally favorable, this study aims to compare the long-term outcomes of patients treated with monobloc versus modular implants. **Methods**: The medical records of all the patients who underwent RHA at a level I trauma center between 2000 and 2011 were retrospectively reviewed. Patients who were available for follow-up were invited for reassessment, which included physical examination, questionnaires for the assessment of elbow pain and function, and follow-up radiographs. **Results:** Out of 35 patients who had RHA, 13 (37%) had a monobloc prosthesis and 22 (63%) had a modular prosthesis. Out of the patients that could be traced, 4 patients from the monobloc group and 10 patients from the modular group agreed to participate in the study. The mean follow-up time was 15 years in the monobloc group and 12.4 years in the modular group. Patients in the modular group demonstrated superior functional outcomes compared to the monobloc group, with statistically significant improvements in MEPS and DASH scores and a non-significant trend towards better ASES scores and VAS scores. Physical examination revealed a decline in function in the operated arm for both groups, with statistically significant differences favoring the modular group in elbow flexion and extension. Radiographic analysis showed varying degrees of implant loosening, with the modular group exhibiting less loosening compared to the monobloc group. Mild degenerative changes and heterotopic ossification were also observed, predominantly in the modular group. **Conclusions:** The results suggest that modular implants offer superior functional outcomes compared to monobloc implants. The modular group showed statistically significant improvements in elbow flexion and extension. These findings indicate that modular implants may be a more favorable option for enhancing patient outcomes. Further research with larger sample sizes is recommended to confirm these trends and to better understand the long-term benefits of modular implants.

## 1. Introduction

The elbow joint is a hinge joint composed of three bones—the ulna, radius, and humerus—with articulations between them. Any fracture involving one or all of these bones can disrupt elbow function, which is crucial for the efficient movement of the forearm and hand [1]. Radial-head fractures (RHFs) account for up to one-third of all elbow fractures. These fractures can occur in isolation or in combination with other elbow fractures and ligament injurie [2].

The radial head is a secondary stabilizer of the elbow and plays an important role in stabilizing the elbow against valgus stress, especially in conjunction with injury or the dysfunction of the medial collateral ligament, which is the primary stabilizer against valgus force [2,3]. In the vast majority of comminuted RHFs, there is soft tissue injury, including damage to the medial collateral ligament. In these situations, it is recommended to replace the radial head with a prosthesis to maintain joint stability [4,5].

RHF typically results from a fall on an outstretched hand with the elbow partially flexed and in pronation [6]. According to the Mason classification, RHFs are divided into three types based on radiographic findings: type 1—a non-displaced RHF (or small marginal fractures); type 2—partial articular fractures with displacement (>2 mm); and type 3—a comminuted fracture involving the entire radial head [7]. Type 4 was subsequently incorporated following the refinement of the original Mason classification: an RHF concurrent with elbow dislocation [8]. Hotchkiss further refined this classification, with similar divisions: non-displaced or minimally displaced fractures (Type I), displaced fractures requiring fixation (Type II), and comminuted fractures often needing prosthetic replacement (Type III) [9].

Each fracture type has an accepted treatment approach. Type 1 fractures are usually treated conservatively with early mobilization. Type 2 fractures with displacement greater than 2 mm that interfere with forearm rotation are typically treated surgically with ORIF. Type 3 fractures are comminuted and often require surgical treatment. This may involve radial-head excision, with or without prosthetic replacement [10].

## 2. Types of Prostheses

Radial-head prostheses are primarily used to treat irreparable RHFs. These prostheses come in several designs. In the past, silicone prostheses were used as a replacement for the radial head, but their use was discontinued after it was discovered that they did not provide sufficient joint stability. Mechanical failures of the implant, including wear and fragmentation, led to the development of synovitis and intra-articular damage [11,12].

Current prosthesis designs were developed to address the problems associated with silicone implants. There are different types of prostheses, including monobloc designs, where the head and neck of the prosthesis are constructed as a single unit in a limited number of sizes. Long-term results of one monobloc prosthesis were published in 2001. The researchers advocated for the use of this prosthesis in cases where joint instability was discovered during surgery after radial-head removal [2]. Sixteen out of twenty patients in the study group demonstrated good to excellent results at an average follow-up of 12 years after RHA with this prosthesis.

Anatomical studies have defined the dimensions of the proximal radius and shown that there is not always a size match between the radial neck and head. Therefore, the need arose for a prosthesis composed of separate head and neck components of varying sizes to allow for more precise anatomical and biomechanical matching to the original radial head [13]. The modular prosthesis offers a range of diameters and thicknesses for the radial head, along with stem implants of varying diameter and height. Any head can be connected to any stem, allowing for dozens of different combinations [14,15].

In the process of inserting the modular prosthesis, the removed radial head serves as a template for measuring the size of the prosthesis, with the aim of finding and fitting a prosthesis that is anatomically as similar as possible to the original radial head. When inserting the prosthesis, it is important to avoid an oversized radial head, which can create high pressure and lead to accelerated cartilage wear in the capitellum area and elbow pain—a phenomenon known as “overstuffing.” An additional theoretical advantage of the modular prosthesis is the ability to insert the stem separately from the prosthetic head, which reduces the extent of surgical exposure and decreases damage to soft tissues and joint ligaments [16].

Results from short- and medium-term studies following RHA cases have shown excellent elbow function in most patients, without joint instability or the need for prosthesis removal due to loosening or infection [17,18]. There is a lack of studies reporting long-term results with emphasis on prosthesis loosening and joint arthritis. Further research is needed on this topic. In the current literature review, no studies comparing non-modular (monobloc) prostheses to modular prostheses were found.

The aim of this study is to evaluate the differences in treatment outcomes between patients who underwent RHA with a monobloc prosthesis versus those who received a modular prosthesis, and to determine if there is an advantage to the more advanced modular prosthesis. The research hypothesis is that the results of RHA with a modular prosthesis will be better than those with a non-modular (monobloc) prosthesis in terms of the function and stability of the operated elbow, as the modular prosthesis is anatomically more adaptable to the removed radial head.

## 3. Methods

The study received approval from the institutional Helsinki Committee (No. 0628-13-TLV). This retrospective, single-center study included adult patients (over 18 years old) who underwent RHA surgery between 2000 and 2011. Patients with pre-existing neurological conditions, multi-trauma (including head injuries), and prior upper limb surgeries that could affect the RHA outcomes were excluded from the study. The baseline demographic characteristics of the patients were obtained from the electronic medical records. These characteristics included age, gender, indication for surgery, associated injuries and need for surgical repair of additional elements, type of implant, post-operative complications and whether a revision surgery was performed.

For the purpose of drawing conclusions and processing data, patients were divided into two groups based on the type of implant inserted: modular or monobloc. Eligible patients were invited for an examination that included a physical examination of elbow range of motion and an assessment of hand and finger grip strength test using a Jamar+ Pinch dynamometer (Patterson Medical, Illinois, USA). All participants completed several questionnaires to assess the function of the operated elbow. Subsequently, lateral and anteroposterior (AP) X-rays of both the operated and healthy elbows were analyzed for each examined patient. The parameters examined include degenerative changes in the joint, the formation of heterotopic ossification, and the degree of lucency around the implant (none, mild, moderate, severe). In the follow-up assessments, patients were evaluated by an independent orthopedic surgeon with a subspecialty in hand surgery. This evaluator was not involved in the surgical procedures, ensuring objectivity in the clinical assessments. All evaluations were conducted in accordance with standardized protocols to minimize bias and maintain consistency in data collection. The study was conducted following the STROBE (Strengthening the Reporting of Observational Studies in Epidemiology) guidelines. (Appendix A)

## 4. Evaluation of Treatment Outcomes

Treatment outcomes should be determined based on functional assessment questionnaires, physical examination, and pain evaluation. Assessment metrics range from simple questionnaires regarding patient satisfaction and ability to return to work to complex scoring methods that combine multiple parameters such as pain, daily activities, and measures based on objective physical examination. In general, outcome measures should be relevant to patients, easy to use, reliable, valid, and responsive to clinical changes [19,20].

The Disabilities of the Arm, Shoulder and Hand (DASH) questionnaire [21]: Introduced in 1996, it was designed to assess one or more disorders in the upper limb. The questionnaire can be used to evaluate functional problems in any area of the upper limb and has been proven valid and responsive to changes compared to other specific measures in the upper limb. The questionnaire consists of 30 core questions and 8 additional optional questions assessing work and sports/performing arts activities.

The American Shoulder and Elbow Surgeons (ASES) questionnaire [22]: Developed in 1999, it was designed to measure elbow function. The questionnaire consists of two parts: the first comprises 19 questions related to the patient’s assessment of pain, function, and satisfaction with surgery, while the second part consists of 38 items evaluating joint motion, strength, and stability, which are completed by the physician. Pain has the greatest impact among the questionnaire components.

The Mayo Clinic Performance Index for the Elbow (MEPS) [23]: Developed in 1992, it was originally intended to evaluate surgical results after total elbow replacement. This questionnaire is based on four variables: pain, elbow range of motion, joint stability, and the patient’s daily activities. Each variable receives a score based on the physician’s assessment. The raw score is divided into four categories: poor (0–59), fair (60–74), good (75–89), and excellent (90–100). The raw score has a better correlation with other methods compared to the categorical division.

Pain assessment using the Visual Analog Scale (VAS): The VAS is a tool for measuring the patient’s subjective pain intensity. It typically consists of a scale ranging from “no pain at all” to “unbearable pain” [24].

## 5. Statistical Methods

Categorical variables were compared using the Chi-squared test or Fisher’s exact test, while continuous variables were compared using the Wilcoxon signed-rank test and Friedman’s two-way analysis of variance by ranks. Statistical significance was set at a *p*-value < 0.05. Categorical variables are expressed as count (percentage), while continuous variables are expressed as mean ± standard deviation. Statistical analysis was performed using IBM SPSS statistics (version 29.0.1).

## 6. Results

A total of 35 patients who underwent RHA with a prosthesis were identified at the Tel Aviv Sourasky Medical Center between 2000 and 2011. Of these, 13 (37%) received a Corin^®^ monobloc implant (Corin Group PLC, Cirencester, UK)) and 22 (63%) received a modular Evolve implant (Wright Medical Technology, Arlington, TN, USA). After excluding patients who declined participation, four patients remained in the monobloc group and ten remained in the modular group. The mean follow-up period was 15 years for the monobloc group and 12.4 years for the modular group. No revision surgeries were performed during the follow-up period. Demographic data, including implant type, concomitant procedures, and early and late complications, are presented in Table 1. There were no statistically significant age differences between groups or between examined and non-examined patients within each group (Table 2 and Table 3).

**Figure 1:**
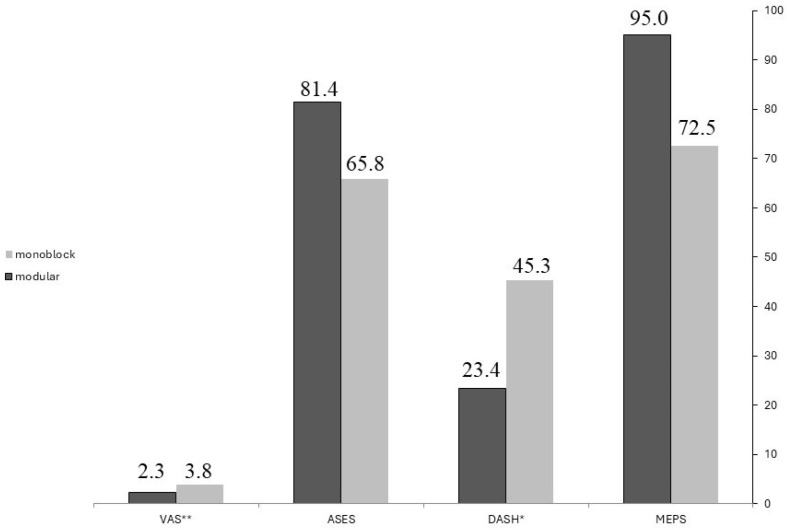
The average mean scores in the function and pain questionnaires between the two study groups. * A low score on the DASH questionnaire indicates a higher functional outcome; ** A low score on the VAS questionnaire indicates a lower pain level. MEPS; The Mayo Clinic Performance Index for the Elbow, ASES; The American Shoulder and Elbow Surgeons questionnaire, DASH; The Disabilities of the Arm, Shoulder and Hand, VAS; Visual Analog Scale.

Patients in the modular group demonstrated superior functional outcomes compared to the monobloc group, with statistically significant better results in MEPS (*p =* 0.014) and DASH (*p =* 0.07) scores. A non-significant trend towards improved function was observed in the ASES scores (*p =* 0.58) for the modular group. Additionally, the modular group showed a non-significant trend towards lower pain levels, as evidenced by lower VAS scores (*p =* 1.00) (Table 4, Figure 1).

Physical examination revealed decreased performance in most variables between the operated and healthy arms in both groups, indicating functional decline in the operated side regardless of implant type (Table 5). A comparison between groups showed statistically significant differences favoring the modular group in elbow flexion (*p =* 0.095, *p =* 0.054) and extension (*p =* 0.07, *p =* 0.106). No significant differences were found in other examined parameters (Table 6).

**Figure 2:**
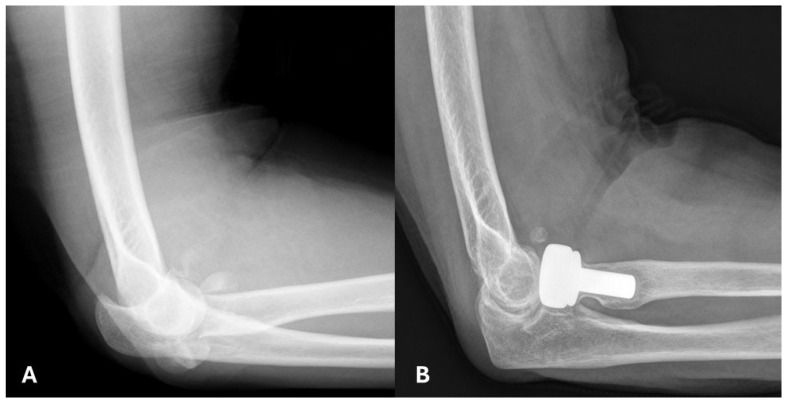
63-year-old patient with a comminuted fracture of the radial head, who underwent a modular radial-head arthroplasty. (**A**) Lateral radiograph of the right elbow, before the operation in 2009. (**B**) Lateral radiograph in 2019, showing heterotopic ossification that does not affect the joint.

**Figure 3:**
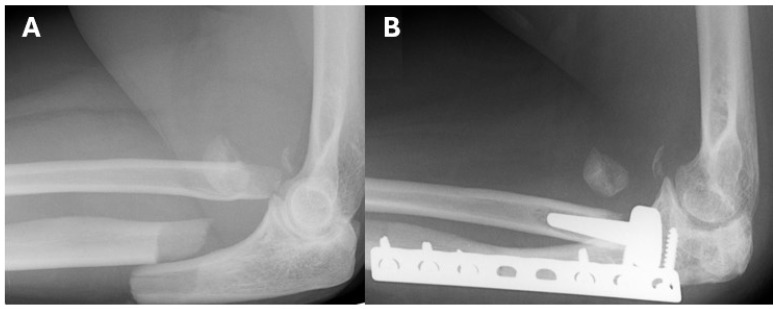
43-year-old female patient with a comminuted fracture of the radial head and a proximal ulna fracture, who underwent fixation of the ulnar fracture with a plate and screws, and implantation of a monobloc radial-head prosthesis. (**A**) Lateral radiograph of the right elbow before surgery in 2007. (**B**) Lateral radiograph in 2018 .In both radiographs (**A**,**B**), fragments of the radial head that were left during surgery can be seen.

Radiographic analysis of all 14 patients showed varying degrees of implant loosening. In the modular group, eight patients (80%) showed no signs of loosening, one (10%) had mild loosening, and one (10%) had moderate loosening. In the monobloc group, all four patients exhibited loosening, with three (75%) showing mild and one (25%) showing moderate loosening. Mild degenerative changes were observed in two (20%) patients in the modular group and one (25%) patient in the monobloc group. Heterotopic ossification was noted in three patients (30%) in the modular group, none of which were functionally limiting, while no cases were observed in the monobloc group (Figure 2 and Figure 3).

## 7. Discussion

Short- and medium-term research outcomes following RHA have demonstrated excellent elbow function in the majority of patients, without joint instability or the need for implant removal due to loosening or infection [6,25,26]. RHA in cases of comminuted RHFs accompanied by ligamentous injury restores joint stability, with a minimal loss of range of motion and strength [3]. There is a paucity of studies reporting long-term outcomes, particularly regarding implant loosening and joint arthritis. The current literature reviews have not identified studies comparing non-modular (monobloc) implants with modular implants.

This study compared clinical outcomes between patients who underwent RHA with monobloc implants and those who received modular implants, aiming to determine if the latter are superior in terms of functional outcomes, including range of motion, strength, and stability. Upon reviewing the results, the modular implant may have advantages over the monobloc implant, manifesting in improved daily function and reduced pain levels. Regarding elbow movements, we demonstrated a statistically significant advantage favoring the modular implant in flexion and extension. The mean differences in these movements between the operated and healthy limbs were smaller in the modular group, indicating the better function of the operated limb and achievement of ranges similar to the healthy elbow in cases where a modular implant was used. For other elbow movements and grip and pinch strength measurements, we were unable to demonstrate an advantage for a specific implant type. Radiographs showed no implant loosening in any of the patients.

The radial head is crucial for elbow stability during varus and valgus loading, as well as for the stability of both the humeroradial and distal radioulnar joints. Consequently, radial-head resection is no longer recommended due to complications such as wrist degeneration, persistent instability, and reduced muscle strength [27].

RHA is indicated for irreparable RHFs associated with elbow instability. While most reported clinical outcomes are from short-term follow-ups, results are generally favorable [28].

Long-term studies on RHA have demonstrated efficacy in restoring elbow stability and preventing complications with promising results [17]. However, challenges remain. Sun et al.’s meta-analysis provided valuable insights into the outcomes of radial-head fracture treatments. They found a 16.7% revision rate for RHA in complex fractures, primarily due to prosthesis oversizing and subluxation. Comparatively, ORIF showed a slightly higher revision rate of 20.1% for similar fracture types. Although ORIF’s revision rate was higher, the difference was not statistically significant. These findings highlight two crucial points: the importance of accurate prosthesis sizing in RHA to minimize complications, and the comparable effectiveness of both RHA and ORIF in managing complex radial-head fractures. These findings highlight the ongoing debate regarding optimal treatment strategies for complex radial-head fractures [29]. Katthagen et al. [30] reported positive early outcomes for monobloc implants in complex elbow injuries, with low short-term removal rates. They found that medial collateral ligament reconstruction may be unnecessary when the radial column is restored. Various prosthesis types show similar short-term results, with monobloc metallic prostheses offering easy implantation and removal.

The longevity of radial-head prostheses is closely tied to maintaining a near-native radiocapitellar relationship. As the use of RHA becomes more widespread, there is a growing concern about the long-term impact on the capitellar surface. Van Riet et al.’s [31] findings highlight the complexity of this issue. They observed that capitellar erosion, while common in failed RHA cases, was not prevalent in overlengthened RHAs. This suggests that factors other than prosthesis height play a role in capitellar wear. Heifner et al.’s study provides further insight into optimizing RHA outcomes [16]. They found that a press-fit radial-head prosthesis aligned with the forearm’s rotational axis produced capitellar pressures more similar to native conditions compared to non-aligned prostheses. This indicates that anatomic alignment could be key to improving the long-term durability of RHAs by optimizing wear properties.

These studies underscore the importance of not only proper sizing but also precise alignment in RHA. They suggest that achieving a biomechanically sound reconstruction that closely mimics natural joint mechanics may be crucial for minimizing complications and extending prosthesis lifespan.

Shimura et al. [32] compared loose-fit and press-fit stems in RHA for comminuted RHFs in 32 patients. They found no significant differences in clinical outcomes or reoperation rates between the two implant concepts. However, periprosthetic osteolysis occurred more frequently in the press-fit group. Despite current asymptomatic status in patients with osteolysis, the authors recommend careful long-term follow-up.

In their review from 2015, Delclaux et al.’s [33] found no significant differences between radial-head prosthesis types for acute trauma or fracture sequelae treatment. They noted several common complications, including loosening in both cemented and uncemented prostheses, pain and stiffness often resulting from oversized components or joint overstuffing, and instability in complex trauma cases involving lateral collateral ligament and coronoid fractures. The review also highlighted the frequent occurrence of osteoarthritis with long-term follow-up. These findings emphasize the importance of proper prosthesis selection and sizing, as well as the careful management of associated injuries in complex cases.

The current study’s findings suggest that modular radial-head implants have an advantage over monobloc implants in most parameters examined. This finding may be explained by the study’s fundamental assumption that the modular implant is better anatomically adapted to the original radial head and causes fewer changes around the implant over time. It is important to note that efforts were made to minimize information bias through up-to-date functional assessments. We employed multiple functional and pain evaluation questionnaires that are relevant to patients, easy to use, reliable, valid, and responsive to clinical changes. Concurrently, we conducted objective physical examinations by the same examiner for all patients.

However, the study’s findings should be interpreted considering its limitations. The retrospective nature of the study presents a potential limitation, as it may introduce selection bias and limit the ability to control confounding factors. The most prominent limitation is the small sample size, resulting from a lack of response among the group of patients identified as suitable for study participation, particularly among those who received monobloc implants. Furthermore, in the past, there was an increased tendency to excise the radial head without replacing it with a prosthesis, consequently limiting the initial group of patients who underwent RHA. Another potential limitation of this study is the difference in follow-up duration between the two groups (15 years vs. 12.4 years), which could influence outcomes related to complications or implant loosening. However, given that both groups had a follow-up period of over 10 years, it is likely that any significant loosening would have been observed within this timeframe. The difference in follow-up length is due to the earlier use of monobloc implants before the introduction of modular implants—a factor beyond our control. We believe that additional prospective studies including larger groups and comparing both implant types are necessary to conclusively determine if one implant is superior to the other.

## 8. Conclusions

The results suggest that modular implants may offer superior functional outcomes compared to monobloc implants. Despite a general decline in physical function in the operated arm across both groups, the modular group showed statistically significant improvements in elbow flexion and extension. These findings indicate that modular implants may be a more favorable option for improving patient outcomes, particularly in terms of range of motion and overall functionality. Clinically, this could influence the choice of implant in favor of modular systems, especially for patients requiring long-term functional recovery. Further research with larger sample sizes is recommended to confirm these trends, explore the potential long-term benefits of modular implants, and assess their impact on complication rates and patient satisfaction.

## Figures and Tables

**Table 1:** Patients’ demographics.

Type	Pat. No.	Gender	Age (Years)	Pre-op Diagnosis	Affected Side	Dominant Hand Y = Yes, N = No	Type of Implant	Primary Surgery Besides Radial-Head Arthroplasty	Early Onset Complains	Late Onset Complains	Follow-Up (Years)
Modular	1	F	63	Terrible triad	R	Y	EVOLVE STD stem 7.5 + 4. STD head 20	Annular Ligament + LUCL Suture			12
	2	M	54	Terrible triad	R	Y	EVOLVE STD stem 6.5 + 4. STD head 22 + 4	Resection of heterotopic ossification + MCL posterior band, Reconstruction of colateral ligament	Numbness in fingers, ulnar aspect.		12
	3	F	69	Mason 3	R	Y	EVOLVE STD stem 6.5. STD head 18		Pain in flexion		13
	4	F	49	Terrible triad, Gustilo 1	R	Y	EVOLVE STD stem 7.5 +2 STD head 22			Numbness in fingers 4,5	12
	5	M	73	Mason 3, Monteggia fracture	L	N	EVOLVE STD stem 6.5 +2 STD head 24	ORIF of proximal ulna with locking plate			12
	6	M	32	Mason 3 + posterior dislocation	L	N	EVOLVE STD stem 7.5 STD head 24	LCL reconstruction		Unstable Elbow—PLRI, Clicks	13
	7	F	54	Mason 3	R	Y	EVOLVE STD stem 6.5. STD head 18 + 2	EDC+ Capsule + Annular ligament repair			13
	8	F	32	Mason 3 + dislocation	L	N	EVOLVE STD stem 4.5. STD head 18 + 2			Numbness in forearm	13
	9	M	15	Mason 3 + Olecranon and Coronoid fractures	L	N	EVOLVE STD stem 7.5 STD head 22	ORIF of the Olecranon with locking plate	Incomplete neuropraxia of Ulnar nerve		11
	10	F	61	Mason 3 + Olecranon and Coronoid fractures	L	N	EVOLVE STD stem 4. STD head 20	Reduction of the olecranon			13
Monobloc	11	F	43	Mason 3, Monteggia fracture	L	N	CORIFIX, MEDIUM THIN	ORIF of proximal ulna with locking plate		Numbness in fingers, ulnar aspect	12
	12	F	67	Mason 3 + dislocation + Coronoid fracture Type1	R	Y	CORIFIX, MEDIUM THIN	Suture of the Coronoid		Creaking + locking of the elbow	17
	13	F	71	Mason 3 + dislocation	R	Y	CORIFIX SMALL THIK				16
	14	F	71	Terrible triad	L	N	CORIFIX SMALL THIN	Refixation of the anterior capsule			15

**Table 2:** Age and gender distribution of all patients at the time of surgery according to the type of implant. SD: standard deviation.

		Age (Surgery)						Gender			
		Mean	SD	Median	N	*p*-Value (Median Test)	*p*-Value (Whitney U Test)	M		F	
								N	Row N %	N	Row N %
Type	Monobloc	55.8	17.4	52.0	13	0.756	0.468	5	38.5%	8	61.5%
	Modular	50.7	17.4	51.5	22			11	50.0%	11	50.0%

**Table 3:** Age and gender distribution of the patients tested and not tested in the final study, according to the type of implant. SD: standard deviation.

			Age (Surgery)						Gender			
			Mean	SD	Median	N	*p*-Value (Median Test)	*p*-Value (Whitney U Test)	M		F	
									N	Row N %	N	Row N %
Type	Monobloc	Not tested	52.7	18.6	52.0	9	0.217	0.503	5	55.6%	4	44.4%
		Tested	63.0	13.5	69.0	4			0	0.0%	4	100.0%
	Modular	Not tested	51.1	17.3	47.5	12	0.670	0.821	7	58.3%	5	41.7%
		Tested	50.2	18.5	54.0	10			4	40.0%	6	60.0%

**Table 4:** Questionnaires for evaluating the function and pain of the operated elbow. Comparison of scores between the two study groups. MEPS; The Mayo Clinic Performance Index for the Elbow, ASES; The American Shoulder and Elbow Surgeons questionnaire, DASH; The Disabilities of the Arm, Shoulder and Hand, VAS; Visual Analog Scale.

		MEPS				*p*-Value (Median Test)	*p*-Value (Whitney U Test)
		Mean	SD	Median	N		
Type	Monobloc	72.5	17.1	75.0	4	0.085	0.014
	Modular	95.0	7.1	100.0	10		
		ASES				*p*-value (Median test)	*p*-value (Whitney U test)
		Mean	SD	Median	N		
Type	Monobloc	65.8	18.9	70.5	4	0.580	0.142
	Modular	81.4	11.8	82.0	10		
		DASH				*p*-value (Median test)	*p*-value (Whitney U test)
		Mean	SD	Median	N		
Type	Monobloc	45.3	18.9	41.5	4	0.070	0.106
	Modular	23.4	22.2	16.0	10		
		VAS				*p*-value (Median test)	*p*-value (Whitney U test)
		Mean	SD	Median	N		
Type	Monobloc	3.8	3.2	3.5	4	1.00	0.304
	Modular	2.3	2.1	3.0	10		

**Table 5:** Physical examination comparison between the operated hand and the healthy one within each of the groups.

			Affected Hand					Unaffected Hand						Tests	
Type	Physical Examination	N	Minimum	Maximum	Median	Mean	Std. Deviation	Minimum	Maximum	Median	Mean	Std. Deviation	Difference	Related Samples Wilcoxon Signed Rank Test	Related Samples Friedman’s Two-Way Analysis of Variance Be Ranks
Monobloc	Extension	4	35.0	25.0	30.0	30.0	5.8	10.0	−5.0	7.5	5.0	7.1	25.00	0.068	0.046
	Flexion	4	110.0	140.0	130.0	127.5	13.2	140.0	145.0	142.5	142.5	2.9	−15.00	0.068	0.046
	Supination	4	30.0	90.0	70.0	65.0	28.0	90.0	90.0	90.0	90.0	0.0	−25.00	0.109	0.083
	Pronation	4	20.0	90.0	77.5	66.3	31.5	80.0	90.0	87.5	86.3	4.8	−20.00	0.109	0.083
	Pinch strength (kg)	4	2.5	5.0	5.0	4.4	1.3	2.0	6.0	5.5	4.8	1.9	−0.38	0.276	0.564
	Grip strength (kg)	4	2.0	18.0	11.5	10.8	6.6	3.0	20.0	15.5	13.5	7.9	−2.75	0.102	0.083
Modular	Extension	10	40.0	−10.0	10.0	11.5	16.7	25.0	−20.0	0.0	0.5	13.2	11.00	0.084	0.096
	Flexion	10	105.0	145.0	135.0	133.5	11.3	135.0	145.0	140.0	138.5	3.4	−5.00	0.066	0.046
	Supination	10	0.0	90.0	70.0	61.5	30.6	60.0	90.0	90.0	84.5	9.6	−23.00	0.018	0.008
	Pronation	10	−15.0	90.0	65.0	60.0	30.2	70.0	90.0	85.0	84.0	6.6	−24.00	0.024	0.034
	Pinch strength (kg)	10	3.0	11.0	5.5	6.3	2.7	3.0	15.0	5.5	7.0	3.6	−0.75	0.176	0.180
	Grip strength (kg)	10	10.0	32.0	21.5	21.5	7.4	16.0	47.0	23.0	25.1	10.4	−3.60	0.104	0.180

**Table 6:** Comparison between the groups in the average differences between healthy and operated hands.

Type	Extension Difference				*p*-Value (Median Test)	*p*-Value (Whitney U Test)
	Mean	SD	Median	N		
Monobloc	−25.00	10.80	−22.50	4	0.070	0.106
Modular	−11.00	17.92	−7.50	10		
	Flexion Difference				*p*-value (Median test)	*p*-value (Whitney U test)
	Mean	SD	Median	N		
Monobloc	15.00	10.80	12.50	4	0.095	0.054
Modular	5.00	9.43	0.00	10		
	Supination Difference				*p*-value (Median test)	*p*-value (Whitney U test)
	Mean	SD	Median	N		
Monobloc	25.00	27.99	20.00	4	1.000	0.945
Modular	23.00	25.73	17.50	10		
	Pronation Difference				*p*-value (Median test)	*p*-value (Whitney U test)
	Mean	SD	Median	N		
Monobloc	20.00	30.28	7.50	4	0.580	0.733
Modular	24.00	32.64	20.00	10		
	Pinch Strength Difference				*p*-value (Median test)	*p*-value (Whitney U test)
	Mean	SD	Median	N		
Monobloc	0.38	0.75	0.50	4	1.000	1.000
Modular	0.75	1.69	0.00	10		
	Grip strength Difference				*p*-value (Median test)	*p*-value (Whitney U test)
	Mean	SD	Median	N		
Monobloc	2.75	4.19	1.00	4	0.559	0.539
Modular	3.60	7.92	0.00	10		

## Data Availability

The data presented in this study are available on request from the corresponding author due to ethical reasons.

## List of Abbreviations

RHF	Radial-Head Fractures
MEPS	Mayo Clinic Performance Index for the Elbow
DASH	Disabilities of the Arm, Shoulder and Hand
ASES	American Shoulder and Elbow Surgeons
VAS	Visual Analog Scale
ORIF	Open Reduction and Internal Fixation
AP	Anteroposterior
EDC	Extensor Digitorum Communis
LUCL	Lateral Ulnar Collateral Ligament
SD	Standard Deviation

## Appendix A

**Table A1:** STROBE (Strengthening the Reporting of Observational Studies in Epidemiology) Guidelines Checklist.

	Item No.	Recommendation	Page No.
Title and abstract	1	(a) Indicate the study’s design with a commonly used term in the title or the abstract	1
		(b) Provide in the abstract an informative and balanced summary of what was done and what was found	2
Introduction			
Background/rationale	2	Explain the scientific background and rationale for the investigation being reported	4
Objectives	3	State specific objectives, including any prespecified hypotheses	5–6
Methods			
Study design	4	Present key elements of study design early in the paper	7
Setting	5	Describe the setting, locations, and relevant dates, including periods of recruitment, exposure, follow-up, and data collection	7
Participants	6	(a) Give the eligibility criteria, and the sources and methods of selection of participants. Describe methods of follow-up	7
		(b) For matched studies, give matching criteria and number of exposed and unexposed	
Variables	7	Clearly define all outcomes, exposures, predictors, potential confounders, and effect modifiers. Give diagnostic criteria, if applicable	7–8
Data sources/measurement	8 *	For each variable of interest, give sources of data and details of methods of assessment (measurement). Describe comparability of assessment methods if there is more than one group	8
Bias	9	Describe any efforts to address potential sources of bias	8–9
Study size	10	Explain how the study size was arrived at	9
Quantitative variables	11	Explain how quantitative variables were handled in the analyses. If applicable, describe which groupings were chosen and why	9
Statistical methods	12	(a) Describe all statistical methods, including those used to control for confounding	9
		(b) Describe any methods used to examine subgroups and interactions	
		(c) Explain how missing data were addressed	
		(d) If applicable, explain how loss to follow-up was addressed	
		(e) Describe any sensitivity analyses	
Results			
Participants	13 *	(a) Report numbers of individuals at each stage of study—eg numbers potentially eligible, examined for eligibility, confirmed eligible, included in the study, completing follow-up, and analysed	
		(b) Give reasons for non-participation at each stage	10–18
		(c) Consider use of a flow diagram	
Descriptive data	14 *	(a) Give characteristics of study participants (eg demographic, clinical, social) and information on exposures and potential confounders	10–18
		(b) Indicate number of participants with missing data for each variable of interest	
		(c) Summarise follow-up time (eg, average and total amount)	
Outcome data	15 *	Report numbers of outcome events or summary measures over time	10–18
Main results	16	(a) Give unadjusted estimates and, if applicable, confounder-adjusted estimates and their precision (eg, 95% confidence interval). Make clear which confounders were adjusted for and why they were included	10–18
		(b) Report category boundaries when continuous variables were categorized	
		(c) If relevant, consider translating estimates of relative risk into absolute risk for a meaningful time period	
Other analyses	17	Report other analyses done—eg analyses of subgroups and interactions, and sensitivity analyses	NA
Discussion			
Key results	18	Summarise key results with reference to study objectives	19
Limitations	19	Discuss limitations of the study, taking into account sources of potential bias or imprecision. Discuss both direction and magnitude of any potential bias	21–22
Interpretation	20	Give a cautious overall interpretation of results considering objectives, limitations, multiplicity of analyses, results from similar studies, and other relevant evidence	21–22
Generalisability	21	Discuss the generalisability (external validity) of the study results	21–22
Other information			
Funding	22	Give the source of funding and the role of the funders for the present study and, if applicable, for the original study on which the present article is based	1

* Give information separately for cases and controls in case-control studies and, if applicable, for exposed and unexposed groups in cohort and cross-sectional studies. Note: An Explanation and Elaboration article discusses each checklist item and gives methodological background and published examples of transparent reporting. The STROBE checklist is best used in conjunction with this article (freely available on the Web sites of PLoS Medicine at http://www.plosmedicine.org/, Annals of Internal Medicine at http://www.annals.org/, and Epidemiology at http://www.epidem.com/). Information on the STROBE Initiative is available at www.strobe-statement.org.
